# Positive environmental modification of depressive phenotype and abnormal hypothalamic-pituitary-adrenal axis activity in female C57BL/6J mice during abstinence from chronic ethanol consumption

**DOI:** 10.3389/fphar.2013.00093

**Published:** 2013-07-23

**Authors:** Terence Y. Pang, Xin Du, William A. Catchlove, Thibault Renoir, Andrew J. Lawrence, Anthony J. Hannan

**Affiliations:** Behavioural Neurosciences Division, Florey Institute of Neuroscience and Mental Health, University of MelbourneMelbourne, VIC, Australia

**Keywords:** alcohol, abstinence, depression, environmental enrichment, HPA axis, dexamethasone, GR, pomc1

## Abstract

Depression is a commonly reported co-morbidity during rehabilitation from alcohol use disorders and its presence is associated with an increased likelihood of relapse. Interventions which impede the development of depression could be of potential benefit if incorporated into treatment programs. We previously demonstrated an ameliorative effect of physical exercise on depressive behaviors in a mouse model of alcohol abstinence. Here, we show that environmental enrichment (cognitive and social stimulation) has a similar beneficial effect. The hypothalamic-pituitary-adrenal (HPA) axis is a key physiological system regulating stress responses and its dysregulation has been separably implicated in the pathophysiology of depression and addiction disorders. We performed a series of dexamethasone challenges and found that mice undergoing 2 weeks of alcohol abstinence had significantly greater corticosterone and ACTH levels following a DEX-CRH challenge compared to water controls. Environmental enrichment during alcohol abstinence corrected the abnormal DEX-CRH corticosterone response despite a further elevation of ACTH levels. Examination of gene expression revealed abstinence-associated alterations in glucocorticoid receptor (*Gr*), corticotrophin releasing hormone (*Crh*) and pro-opiomelanocortin (*Pomc1*) mRNA levels which were differentially modulated by environmental enrichment. Overall, our study demonstrates a benefit of environmental enrichment on alcohol abstinence-associated depressive behaviors and HPA axis dysregulation.

## Introduction

One of the biggest impediments to recovery programs for alcohol use disorders is the development of psychological disturbances by patients, such as post-dependent dysphoric syndromes. This is a significant issue to be addressed because the presence of co-morbid psychiatric conditions such as depression and anxiety during abstinence is linked to a greater probability of relapse (Pelc et al., 2002). However, attempts to improve rehabilitation rates are hindered by the uncertainty over the precise causes of abstinence-associated psychopathology. Numerous studies of rodent models have provided evidence that withdrawal from exposure to addictive compounds elicit depression-related behavioral phenotypes (reviewed by Renoir et al., 2012). Not dissimilar, depression-related behavioral changes also feature in rodents withdrawn from alcohol and include anhedonia and helplessness, similar to the major aspects of clinical depression (Rasmussen et al., 2001; Stevenson et al., 2009; Fukushiro et al., 2012; Pang et al., 2013). Several studies have established that the withdrawal phase itself is marked by specific cellular and molecular changes in the brain (Crews et al., 2004; Nixon and Crews, 2004; Aberg et al., 2005; He et al., 2009; Stevenson et al., 2009). Recently, Vendruscolo and colleagues proposed that the phases of acute withdrawal and protracted abstinence are distinct within themselves, marked by differences in hypothalamic-pituitary-adrenal (HPA) axis activity and expression levels of the glucocorticoid receptor (Vendruscolo et al., 2012).

The HPA axis is the key physiological system that regulates circulating levels of adrenal stress hormones (cortisol, corticosterone). Dysregulation of HPA axis activity is separably implicated in the pathology of addiction disorders (see reviews by Sinha, 2008; Picciotto et al., 2010; Picetti et al., 2013) and depression (see reviews by Braquehais et al., 2012; Laryea et al., 2012; Lopresti et al., 2013). Modification of HPA axis signaling can alter drug seeking behavior (Deroche-Gamonet et al., 2003; Pastor et al., 2008; Wang et al., 2008). There have been few examinations of HPA axis regulation in a clinical population of alcoholics or those undergoing rehabilitation. However, the early evidence is that HPA axis activity is dysregulated during alcohol withdrawal. The biphasic nature of withdrawal proposed by Vendruscolo is consistent with the finding that recovering alcoholics have increased levels of cortisol and ACTH levels initially, which typically normalize upon completion of a rehabilitation program (Hundt et al., 2001). While an increase in ACTH levels is indicative of anterior pituitary dysfunction, a recent report indicated increased methylation of the *pomc1* gene promoter region in alcohol dependent patients (Muschler et al., 2010) which translates to suppression of gene expression and a predicted reduction in ACTH levels. The conflicting implications of these studies demonstrate that further work is required to better understand the nature of HPA axis pathology during abstinence from alcohol.

The development of non-pharmacological interventions for the treatment of addiction and depression are highly attractive as simple and low-cost approaches. We previously demonstrated that mice abstinent from alcohol display depression-related behaviors which are ameliorated by engaging in physical exercise (wheel-running) provided during the period of abstinence (Pang et al., 2013). Other groups have reported that environmental enrichment, a paradigm of social and cognitive stimulation, has the capacity for non-drug modification of addiction-related behaviors (Solinas et al., 2008; Chauvet et al., 2009; Nader et al., 2012). Furthermore, environmental enrichment can exert anti-depressive effects on behavior in several models of depression (Pang et al., 2009; Hendriksen et al., 2012; Lehmann et al., 2013; Stuart et al., 2013), possibly through HPA axis modulation (Du et al., 2012). Therefore, we sought to investigate whether environmental enrichment could exert a corrective effect on the depressive phenotype of a mouse model of alcohol abstinence.

## Materials and methods

### Mice

Six-week old female C57BL/6J mice were purchased from Animal Resources Centre (Murdoch, WA, Australia) and housed at the Florey Neuroscience Laboratories (University of Melbourne, VIC, Australia) in a temperature-controlled environment on a 12:12 h light-dark cycle with food and water provided *ad libitum*. All the behavioral studies were conducted at the Integrative Neuroscience Facility (INF). All experiments were approved by the Howard Florey Institute animal ethics committee in accordance with the recommended guidelines set by the National Health and Medical Research Council (NHMRC) of Australia.

### Ethanol self-administration

**Figure 1:**
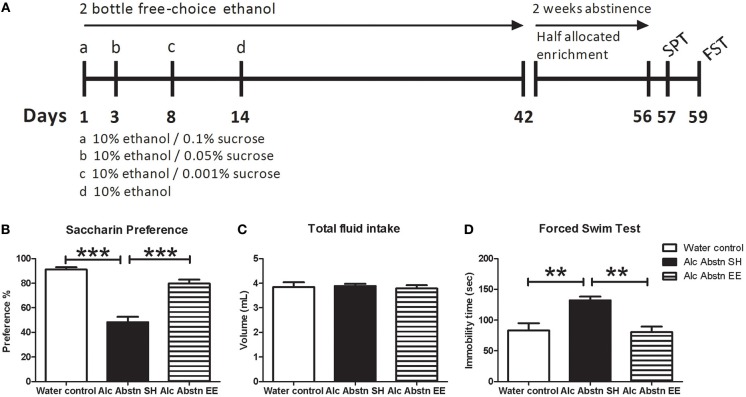
**Environmentally enriched alcohol abstinent mice do not display pro-depressive behaviors**. **(A)** Schematic representation of experimental design leading up to behavioral testing. An identical design was used in the lead up to the DEX combinatorial challenges. **(B)** Reduced saccharin preference associated with alcohol abstinence was not observed in the enriched group. **(C)** Differences in saccharin preference test were not due to change in total fluid consumption. **(D)** Alcohol abstinent mice recorded significantly greater immobility times in the forced-swim test compared to water controls and enriched abstinent mice. Alc Abstn SH: 2 week alcohol abstinence in standard housing conditions; Alc Abstn EE: 2 week alcohol abstinence in environmentally enriched housing conditions. 10 mice per group. 1-way ANOVA followed by *post-hoc* Bonferroni's *t*-test: ^**^p < 0.01; ^***^p < 0.001.

From 8 weeks of age, mice were allowed to self-administer 10% (v/v) ethanol solution (two bottle free-choice) for a period of six weeks as previously published (Pang et al., 2013). An alternative source of untreated water was freely available at all times (see Figure 1A for study schematic). The placements of the ethanol- and water-containing bottles were randomly alternated throughout the experiment to avoid location preference bias. The control group only had access to normal tap water provided in two drink bottles. All mice were single-housed during the first 6 weeks of this study and daily fluid intake was recorded. Consistent with our previous publication (Pang et al., 2013), mice did not differ in total daily fluid intake (data not shown). Ethanol consuming mice showed high preference >85%, averaging 15–18 g/kg alcohol per day. There was no difference in weight gain across the six weeks. After six weeks of free-choice ethanol drinking, the ethanol solution-containing drink bottle was removed for two weeks prior to commencement of behavioral testing. Water-drinking mice were provided a single bottle of water during this period. Mice were randomly allocated to continue being maintained in standard-housing (Alc Abstn SH) or undergo environmental enrichment (Alc Abstn EE). Mice undergoing enrichment were re-grouped 4–6 mice per cage since social stimulation was part of the enrichment paradigm. Enriched mice were housed in larger cages supplemented with shredded paper, tunnels and objects of varying textures and shapes. The configuration of the cage was changed every 3 days. Behavioral testing commenced after 14 days of abstinence; separate cohorts of mice were used for the DEX combinatorial challenges which were conducted on the 15th day of abstinence.

### Saccharin preference test (SPT)

Mice (10 per group) were single-housed over a 12 h overnight period and provided the opportunity to consume 0.1% (w/v) saccharin solution or tap water (Short et al., 2006). The total volume of fluid intake was recorded, and preference ratio was determined by calculating the volume of saccharin solution consumed as a proportion of total fluid intake. Enriched mice were re-grouped in their enrichment cages after the test.

### Forced-swim test (FST)

Mice (10 per group) were individually placed into beakers (17 cm diameter) of water (23–25°C) filled to a depth such that tails would not be in contact with the bottom of the beaker. Each test lasted for a total of 5 mins and was video recorded for subsequent scoring by an experimenter who was blind to treatment and housing conditions of the mice. The total immobility time adopted by each mouse over the final 4 mins of the test was recorded.

### Dexamethasone challenges

The dexamethasone suppression test (DST) involved a single i.p. injection of dexamethasone (DEX) (0.1 mg/kg body weight; Sigma-Aldrich, St. Louis, MO, USA) between 0800–1000H. Six hours later, mice were killed and trunk blood collected for corticosterone analysis. For the DEX-CRH and DEX-ACTH challenges, mice were treated according as per the DST. Six hours after DEX administration, mice received CRH (i.p., 20 mg/kg body weight; Sigma-Aldrich) or ACTH (i.p., 50 μg/100 g body weight; Prospec, Rehovot, Israel). Thirty mins post-CRH/ACTH injections, mice (4–6 per group) were killed and trunk blood collected for corticosterone analysis.

### Quantification of serum corticosterone and ACTH levels

For basal levels of corticosterone, non-stress mice were killed between 0900–1100H for blood collection. Post-stress levels were determined with blood samples collected from mice exposed to 10 mins of forced-swim stress performed between 0900–1100H then killed immediately after. Briefly, mice were killed by cervical dislocation and trunk blood was collected, allowed to coagulate at room temperature for 30 mins before being centrifuged at 1070 rcf for 15 mins. Serum was collected and stored at –20°C until quantification of corticosterone was performed. Corticosterone was quantified using EIA (Cayman Chemical, Ann Arbor, MI, USA) according to the manufacturer's instructions. Serum ACTH levels were determined using a Milliplex Mouse Bone Panel 2A kit (Millipore, St. Charles, MO, USA) as per the manufacturer's recommendations. Samples were read on a Luminex 100 instrument. These analyses were performed by Cardinal Bioresearch (New Farm, QLD, Australia).

### Tissue collection, sample preparation and semi-quantitative real-time PCR

Mice (5–6 per group) were killed via cervical dislocation and brains were removed for microdissection of the relevant regions. Adrenal glands were harvested. All tissue was snap frozen in liquid nitrogen and stored at –80°C. Tissue was disrupted using a bioruptor and RNA was isolated using RNeasy RNA Mini kits (Qiagen, Melbourne, VIC, Australia) according to the manufacturer's instructions. Extracted RNA was stored at –80°C. Sample was reverse transcribed into cDNA using SuperScript®VILO™ cDNA synthesis kit (Invitrogen, Mulgrave, VIC, Australia) according to the manufacturer's instructions. cDNA products were stored at –20°C until further use. cDNA was amplified using the SYBR Green JumpStart Taq Ready Mix (Sigma, Castle Hill, NSW, Australia) based on the manufacturer's instructions [primer sequences are provided in Du et al. (2012)]. Efficiency curves and optimal reaction volumes for all primer pairs were determined. Glucocorticoid receptor (GR, nr3c1) and mineralocorticoid receptor (MR, nr3c2) expression was measured in the hypothalamus and pituitary. CRH expression was measured in the hypothalamus and proopiomelanocotin (POMC1) and dopamine receptor D2 (Drd2) expression was measured in the pituitary gland. Real-time quantitative PCR was carried out using the Applied Biosystems 7500 Fast Real-time PCR system sequence detection software version 1.4 (Applied Biosystems, Foster City, CA, USA). Cyclophilin was used as an endogenous control for the hypothalamus and pituitary analyses. Each sample and housekeeping control was run in duplicate.

### Statistical analysis

Statistical analyses were performed using SPSS statistics 17.0 and graphical data was generated with GraphPad Prism 5.0. Data was analysed with one or two-way analysis of variance (ANOVA) and where a significant difference was detected, followed up with *post-hoc* Bonferroni *t*-tests to determine specific between-group differences. In all cases, the significance level was set at *p* < 0.05.

## Results

### Ethanol abstinence-associated depressive phenotype is corrected by environmental enrichment

#### Saccharin preference test

One-way ANOVA revealed a significant difference in saccharin preference between the groups [*F*_(2, 29)_ = 44.6, *p* < 0.001] (Figure 1B). Standard-housed alcohol abstinent (Alc Abstn) mice had decreased saccharin preference compared to water controls (*p* < 0.001). Environmentally enriched Alc Abstn mice had significantly greater saccharin preference compared to the standard-housed Alc Abstn group (*p* < 0.001), but not different to controls. There was no significant difference in total fluid consumption during the test [*F*_(2, 29)_ = 0.131, *p* = 0.818] (Figure 1C).

#### Forced swim test

One-way ANOVA revealed a significant difference between the groups for total immobility time in the FST [*F*_(2, 29)_ = 10.29, *p* < 0.001] (Figure 1D). *Post-hoc* testing showed that the standard-housed Alc Abstn mice averaged greater immobility times than water controls (*p* < 0.01). Environmentally enriched Alc Abstn mice had significantly reduced FST immobility times compared to the standard-housed group (*p* < 0.01), but not different to controls.

### Ethanol abstinence is associated with abnormal DEX-CRH response

**Figure 2:**
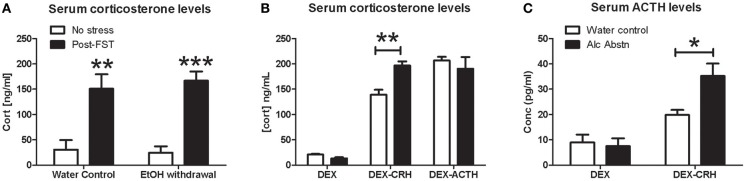
**Alcohol abstinence associated with abnormal DEX-CRH response**. **(A)** No significant difference in serum corticosterone levels at baseline and immediately following forced-swim stress. **(B)** Similar levels of serum corticosterone after DEX and DEX-ACTH challenges. Alcohol abstinent mice had significantly higher corticosterone levels compared to water controls in the DEX-CRH challenge. **(C)** Serum ACTH levels were similar after DEX challenge but alcohol abstinent mice have significantly higher levels of ACTH after the DEX-CRH challenge. 4–6 mice per group. Two or one-way ANOVA followed by *post-hoc* Bonferroni's *t*-test: ^*^p < 0.05, ^**^p < 0.01, ^***^p < 0.001.

Quantification of serum corticosterone levels after forced-swim stress revealed an overall effect of stress (*F*_(1, 14)_ = 45.6, *p* < 0.001) with no difference between the treatment groups [*F*_(1, 14)_ = 0.0652, *p* = 0.80] (Figure 2A). There were no apparent differences in baseline and post-stress corticosterone levels. We examined HPA axis activity in further detail by conducting the dexamethasone suppression challenge in combination with CRH and ACTH. There were no significant differences in serum corticosterone levels between water controls and alcohol abstinent mice following DEX and DEX-ACTH treatments (Figure 2B). However, alcohol abstinent mice had significantly higher serum corticosterone levels compared to water controls following the DEX-CRH challenge (*p* < 0.01). Serum ACTH levels did not differ between the groups after DEX challenge, but was significantly higher in alcohol abstinent mice compared to water controls after DEX-CRH challenge (*p* < 0.05) (Figure 2C).

### Environmental enrichment modifies DEX-CRH response of alcohol abstinent mice

**Figure 3:**
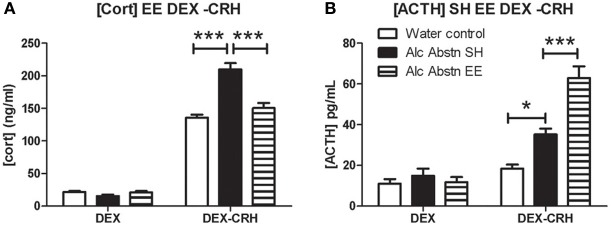
**Environmental enrichment modifies DEX-CRH response of alcohol abstinent mice**. **(A)** Serum corticosterone levels did not differ between the groups in the DEX challenge. In the DEX-CRH challenge, Alc Abstn mice had significantly higher levels of corticosterone compared to water controls and the environmentally enriched abstinent mice. **(B)** The three groups had similar ACTH levels following the DEX challenge. However, following DEX-CRH, enriched Alc Abstn mice responded with even greater levels of ACTH compared to the standard-housed Alc Abstn group. Alc Abstn SH: 2 week alcohol abstinence in standard housing conditions; Alc Abstn EE: 2 week alcohol abstinence in environmentally enriched housing conditions. 6 mice per group. One-way ANOVA followed by *post-hoc* Bonferroni's *t*-test: ^*^p < 0.05; ^***^p < 0.001.

Having found a specific difference in corticosterone response in the DEX-CRH challenge, we repeated the DEX-CRH challenge, and included a group of Alc Abstn mice that had undergone environmental enrichment during the abstinence period. There was no significant difference between the three groups in the DEX challenge [*F*_(2, 17)_ = 2.82, *p* = 0.09] (Figure 3A). However, one-way ANOVA detected a significant difference between the groups in the DEX-CRH challenge [*F*_(2, 17)_ = 26.12, *p* < 0.001]. *Post-hoc* Bonferroni's *t*-test showed that serum corticosterone levels of standard-housed Alc Abstn mice were significantly greater than water controls (*p* < 0.001) and environmentally enriched Alc Abstn mice (*p* < 0.001).

Serum ACTH levels did not differ between the groups in the DEX challenge [*F*_(2, 17)_ = 0.51, *p* = 0.61] (Figure 3B). ACTH levels differed significantly between the groups in the DEX-CRH challenge [*F*_(2, 17)_ = 32.64, *p* < 0.001]. Similar to the data in Figure 2C, standard-housed Alc Abstn mice had higher ACTH levels than water controls (*p* < 0.05). Surprisingly, environmentally enriched Alc Abstn mice had significantly higher levels of ACTH compared to standard-housed Alc Abstn mice (*p* < 0.001).

### Modified gene expression during ethanol abstinence: effects of environmental enrichment

Withdrawal from an acute binge-like ethanol intake is associated with stress hyper-reactivity manifesting as enhanced CORT and ACTH responses to stress (Buck et al., 2011). The findings that Alc Abstn mice respond with significantly higher levels of corticosterone and ACTH in the DEX-CRH challenge is further evidence of HPA axis hyperactivity. To gain a better appreciation of this pathophysiology, we examined the mRNA levels of key regulatory genes involved in HPA axis function, namely the glucocorticoid receptor (GR), mineralocorticoid receptor (MR), crh, pomc1 (the precursor of ACTH) and dopamine 2 receptor (drd2). A previous study had demonstrated the dynamic nature of GR mRNA expression which differs between a state of acute withdrawal (down-regulation) and protracted abstinence (up-regulation) (Vendruscolo et al., 2012). We examined pituitary drd2 gene expression due to evidence of dopamine D2 receptor-mediated regulation of pomc1 mRNA levels (Cote et al., 1986; Pardy et al., 1990).

**Figure 4:**
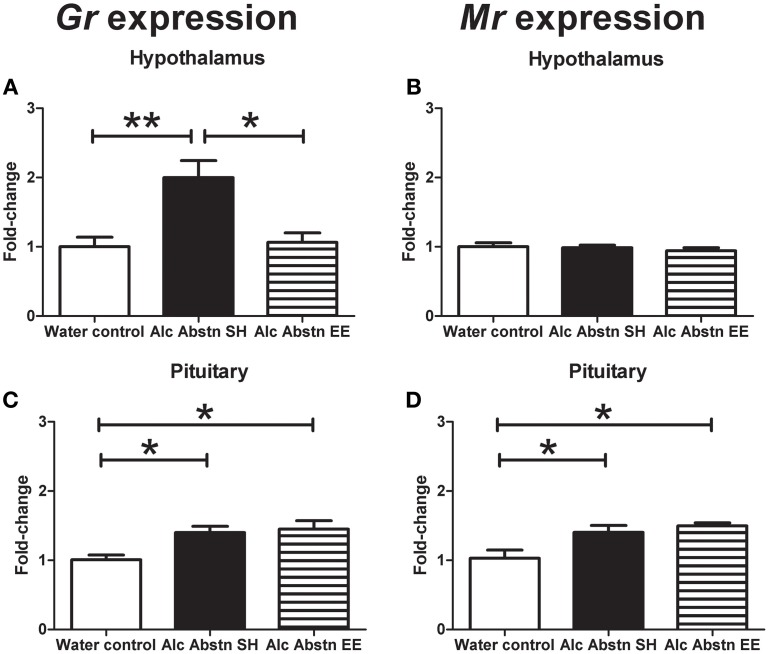
**Differential effects of environmental enrichment on steroid hormone receptors**. **(A)** An up-regulation of GR expression in the hypothalamus of Alc Abstn mice is not observed in the group exposed to enrichment. **(B)** No observable change to hypothalamic MR expression. **(C)** Up-regulation of pituitary GR expression in Alc Abstn mice is maintained in the enriched group. **(D)** In contrast to the hypothalamus, pituitary MR expression was increased with alcohol abstinence, and this persisted in the enriched group. Alc Abstn SH: 2 week alcohol abstinence in standard housing conditions; Alc Abstn EE: 2 week alcohol abstinence in environmentally enriched housing conditions. Five mice per group. One-way ANOVA followed by *post-hoc* Bonferroni's *t*-test: ^*^p < 0.05, ^**^p < 0.01.

In the hypothalamus, GR mRNA levels were significantly different between the groups [*F*_(2, 14)_ = 9.288, *p* = 0.003] (Figure 4A). *Post-hoc* Bonferroni showed that GR gene expression was significantly greater in the standard-housed Alc Abstn group compared to both the water controls (*p* < 0.01) and the environmentally enriched Alc Abstn group (*p* < 0.05). In contrast, there was no significant difference in MR gene expression between the groups [*F*_(2, 14)_ = 0.429, *p* = 0.661] (Figure 4B).

GR mRNA levels were also significantly different between the groups in the pituitary [*F*_(2, 14)_ = 6.389, *p* = 0.013] (Figure 4C). *Post-hoc* testing showed greater MR gene expression in the standard-housed Alc Abstn group (*p* < 0.05) and environmentally enriched Alc Abstn group (*p* < 0.05) compared to water controls. Similarly, but in contrast to the findings in the hypothalamus, pituitary MR mRNA levels significantly differed between the groups [*F*_(2, 14)_ = 6.973, *p* = 0.01] (Figure 4D) with both standard-housed (*p* < 0.05) and environmentally enriched Alc Abstn (*p* < 0.05) groups having higher expression levels compared to the water control group.

**Figure 5:**
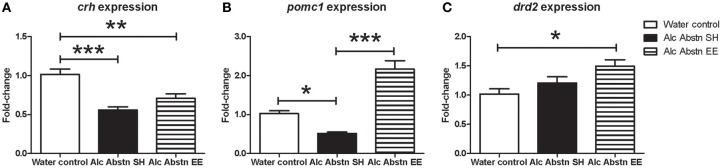
**Differential effects of enrichment on hypothalamic and pituitary gene expression**. **(A)** Hypothalamic *Crh* expression was significantly reduced in the standard-housed Alc abstn group, and unaltered by enrichment. **(B)** Pituitary *pomc1* expression was significantly reduced in the standard-housed Alc Abstn group but was significantly elevated in the enriched group. **(C)** Pituitary *Drd2* expression was not significantly altered by alcohol abstinence. Alc Abstn SH: 2 week alcohol abstinence in standard housing conditions; Alc Abstn EE: 2 week alcohol abstinence in environmentally enriched housing conditions. 5–6 mice per group. 1-way ANOVA followed by *post-hoc* Bonferroni's *t*-test: ^*^p < 0.05, ^**^p < 0.01, ^***^p < 0.001.

*Crh* gene expression was also significantly different between the groups [*F*_(2, 14)_ = 17.72, *p* < 0.001] (Figure 5A). *Post-hoc* testing showed significantly reduced *Crh* mRNA levels in the standard-housed Alc Abstn group (*p* < 0.001) and environmentally enriched Alc Abstn group (*p* < 0.01) compared to water controls.

In the pituitary, *pomc1* gene expression was significantly different between the groups [*F*_(2, 14)_ = 45.78, *p* < 0.001] (Figure 5B). The expression level in standard-housed Alc Abstn mice was 0.51 fold compared to the water control group (*p* < 0.05). *pomc1* gene expression was significantly increased in environmentally enriched Alc Abstn mice when compared to water control (*p* < 0.001) and standard-housed Alc abstn (*p* < 0.001) groups. There was a significant difference in *drd2* gene expression between the groups [*F*_(2, 14)_ = 5.05, *p* = 0.0238] (Figure 5C) with *post-hoc* analysis revealing no significant difference between controls and standard-housed Alc Abstn mice, but a significant elevation in environmentally enriched Alc Abstn mice compared to controls (*p* < 0.05).

## Discussion

Our study has provided further evidence that the expression levels of key centrally-expressed regulators of HPA activity are altered during abstinence from self-administration of alcohol. More specifically, we noted an up-regulation of the two major adrenal steroid receptors in the brain, with the observed effect on GR consistent with previous reports (Vendruscolo et al., 2012). We also observed a region-specific effect of abstinence since MR was up-regulated in the pituitary but remained unaffected in the hypothalamus. Additionally, hypothalamic expression of crh and pituitary expression of pomc1 were down-regulated. The collective data suggest suppression of HPA axis activity during abstinence following chronic alcohol consumption. Given the current evidence implicating HPA axis pathophysiology as a feature of clinical depression, our findings support the hypothesis that dysregulation of the HPA axis is a key event for the development of abstinence-related depression which we have replicated in a mouse model.

### Functional characterization of HPA axis pathology during alcohol abstinence

This is the first study attempting to functionally characterize pathophysiology of the HPA axis during abstinence from alcohol in mice. By performing the DEX challenge in combination with CRH and ACTH administration, we interrogated steroid receptor-mediated suppression of corticosterone levels as well as pituitary and adrenal function. It was important to determine the response to DEX because DEX non-suppression is a reported feature of clinical depression associated with high levels of stress (Fountoulakis et al., 2004) and a smaller suppressive response to DEX has been linked to GR polymorphisms which increase risk for depression (see review by Manenschijn et al., 2009). However no significant difference in basal and post-DEX corticosterone levels between the abstinent and control groups were observed suggesting that suppression of HPA axis activity via down-stream signaling from GR is likely to be normative in abstinent animals. In contrast, we found a significantly greater corticosterone response of abstinent animals compared to controls in the DEX-CRH challenge, matched by an exaggerated elevation of ACTH. These suggest a pathological pituitary response initiated by CRH signaling in abstinent animals. The pathology is likely to be limited to the pituitary since direct stimulation of the adrenals to elicit corticosterone secretion in the DEX-ACTH challenge yielded comparable results.

As far as we are aware, this is the first time an abnormal DEX-CRH response has been reported in a mouse model of alcohol abstinence. However, our findings are consistent with the limited available clinical data. Hundt et al. also performed the DEX-CRH challenge on 19 alcoholic inpatients and reported significantly elevated cortisol and ACTH responses (Hundt et al., 2001). Interestingly, upon completion of the withdrawal program, the DEX-CRH responses of patients were largely normalized. It is important to note that another clinical study has reported that during the acute phase of withdrawal, a pathological DEX-CRH response is limited to increased cortisol levels but not ACTH (Zimmermann et al., 2003). Thus, given that we observed an increased ACTH response in the DEX-CRH test, our study design of 2 weeks abstinence accurately models protracted, not acute, abstinence. The molecular mechanisms involved in the normalization of a pathological DEX-CRH response following protracted alcohol abstinence are presently unknown and will require further investigation. Elucidating the precise signaling pathways involved could contribute to pharmacotherapies which facilitate rehabilitative efforts.

### Environmental enrichment corrects abstinence-related depressive behaviors

The corrective effect of environmental enrichment on saccharin preference and FST immobility suggests that cognitive and social stimulation imparts benefits in ameliorating withdrawal-associated depressive behaviors. Independent groups have demonstrated that environmental enrichment is a modifier of addiction-related neurobiology by preventing the incubation of cocaine craving (Chauvet et al., 2012) and attenuating cocaine seeking behavior (Thiel et al., 2009). Enrichment has also been reported to elicit a blunted ACTH response to stress associated with nicotine withdrawal (Skwara et al., 2012). Our study is the first to demonstrate a benefit of environmental enrichment on the depression-related behavioral phenotype associated with alcohol abstinence and extends our previous work on physical exercise as a potential anti-depressive intervention (Pang et al., 2013).

### Glucocorticoid receptor gene expression is up-regulated during protected alcohol abstinence

Our data also indicated that a variety of molecular regulators of the HPA axis are differentially susceptible to modulation by environmental enrichment. Prior to this study, the regulation of GR gene expression by enrichment in the context of alcohol abstinence had not been investigated. Indeed, despite the positive effects of enrichment in the context of addiction, little is known about the potential molecular mechanisms underlying those effects. Our findings of increased GR gene expression are in agreement with elevated corticosterone concentrations in the brain after a period of ethanol withdrawal (Little et al., 2008). A GR-selective effect of environmental enrichment (sparing MR) in normative mice has previously been reported (Olsson et al., 1994) and this gene-specific effect of enrichment was observed in our examination of the hypothalamus. However, a previous study had found that GR protein levels are decreased after acute (24 h) withdrawal of ethanol (Roy et al., 2002) which is in contrast to the increased GR expression we observed in the hypothalamus and pituitary. This conflicting finding could reflect the differential regulation of the glucocorticoid receptor (both gene and protein levels) during acute withdrawal and after a prolonged period of withdrawal. A more definitive understanding of GR regulation under these different conditions would require future studies that directly compare GR mRNA and protein levels. Roy and colleagues had proposed that diminished GR function in hypothalamus was likely to be the underlying pathology responsible for HPA axis dysfunction during ethanol exposure and withdrawal. While that might be true during the acute phase of withdrawal, our findings together with Vendruscolo et al. (2012) indicate that it is in fact an up-regulation of GR expression during protracted alcohol abstinence that underlies HPA axis pathology. This was further supported by our observation that environmentally enriched abstinent mice had normative levels of GR in the hypothalamus.

### Environmental enrichment corrects abstinence-related abnormal DEX-CRH response and gene expression

It is known that chronic alcohol consumption leads to prolonged activation of the HPA axis, persistent increases in circulating cortisol/corticosterone levels and culminating in dysregulation of *crh* gene expression which itself is a crucial factor in mediating chronic alcohol-related neuroadaptations (see review by Heilig and Koob, 2007). Our finding of decreased hypothalamic *crh* gene expression after protracted abstinence following chronic ethanol consumption is consistent with previous reports (Falco et al., 2009; Silva and Madeira, 2012). However, it is likely that the down-regulation of *crh* is a key pathological change during the process of chronic alcohol consumption which persists once alcohol is withdrawn (Richardson et al., 2008). To date, there has only been one study examining the modulation of *crh* expression by environmental enrichment which reported a non-significant increase in the hypothalamus (Francis et al., 2002). Consistent with that, we did not observe any significant effect of environmental enrichment on *crh* expression in the alcohol abstinent group. One implication of this result is that the beneficial effect of environmental enrichment in correcting the abnormal DEX-CRH response of abstinent mice is downstream of the hypothalamus, and that possibility is supported by our work which described peripheral effects of enrichment on adrenal secretion of corticosterone (Du et al., 2012).

The non-effect of enrichment on hypothalamic *crh* expression is in marked contrast to the surprising up-regulation of pituitary *pomc1* expression, further highlighting the specific nature of enrichment effects on gene expression. A previous study of rats maintained on a 7-week dark-phase ethanol consumption paired with daytime withdrawal reported a suppression of pomc1 mRNA levels by the end of a 3-week gradual ethanol withdrawal procedure (Rasmussen et al., 2000). That finding is consistent with our data on the alcohol abstinent mice which is not surprising given the somewhat similar design of the studies. This is the first report of an enrichment-associated up-regulation of *pomc1* which is consistent with the further elevation of ACTH levels in this group for the DEX-CRH challenge. However, the increased gene expression and greater functional output do not corroborate with the normalization of corticosterone levels following DEX-CRH. At the present time, we are only able to speculate on the mechanisms which could account for this apparent inconsistency. It is possible that the corrective effect of enrichment lies downstream of ACTH, i.e., exposure to environmental enrichment modifies the expression pattern of ACTH receptors located peripherally in the adrenal cortex. This possibility is supported by a study that described enrichment-mediated alterations of the temporal profile of HPA axis activity (Moncek et al., 2004).

### Arginine vasopression as a potential modifier of HPA axis in alcohol abstinence

Another potential modifier of ACTH is the stress-responsive arginine vasopressin (AVP) which is implicated in high-alcohol drinking behavior (Zhou et al., 2011a). AVP is reportedly increased after protracted abstinence from cocaine (Zhou et al., 2011b) but has yet to be investigated thoroughly in the context of alcohol abstinence. Interestingly, naloxone administration (an opioid receptor antagonist commonly used to diminish alcohol craving) has been reported to result in an up-regulation of AVP expression. However, the mechanism of AVP-dependent ACTH release is likely to involve multiple signaling pathways (Perdona et al., 2012). Regulation by AVP could account for our observation of an apparent dissociation between ACTH and corticosterone levels since control-like corticosterone levels were elicited from enriched abstinent mice in the DEX-CRH challenge despite the presence of more exaggerated ACTH levels. Further work will be required to determine if AVP-mediated signaling is involved in imparting the corrective effects of environmental enrichment in the DEX-CRH challenge.

In summary, our study has provided molecular and functional evidence of pituitary pathology in protracted abstinence from alcohol. Environmental enrichment was able to prevent the development of abstinence-associated depression-related behaviors and corrected the pathological DEX-CRH corticosterone response. Further studies investigating the precise molecular mechanisms underlying the benefits of enrichment could uncover novel therapeutic targets to facilitate rehabilitation from alcoholism.

### Conflict of interest statement

The authors declare that the research was conducted in the absence of any commercial or financial relationships that could be construed as a potential conflict of interest.
